# Resveratrol Increases Serum BDNF Concentrations and Reduces Vascular Smooth Muscle Cells Contractility via a NOS-3-Independent Mechanism

**DOI:** 10.1155/2017/9202954

**Published:** 2017-02-05

**Authors:** Michał Wiciński, Bartosz Malinowski, Mateusz M. Węclewicz, Elżbieta Grześk, Grzegorz Grześk

**Affiliations:** Department of Pharmacology and Therapeutics, Faculty of Medicine, Collegium Medicum in Bydgoszcz, Nicolaus Copernicus University, M. Curie 9, 85-090 Bydgoszcz, Poland

## Abstract

Resveratrol is a polyphenol that presents both antineuroinflammatory properties and the ability to interact with NOS-3, what contributes to vasorelaxation. Brain-derived neurotrophic factor (BNDF), a molecule associated with neuroprotection in many neurodegenerative disorders, is considered as an important element of maintaining stable cerebral blood flow. Vascular smooth muscle cells (VSMCs) are considered to be an important element in the pathogenesis of neurodegeneration and a potential preventative target by agents which reduce the contractility of the vessels. Our main objectives were to define the relationship between serum and long-term oral resveratrol administration in the rat model, as well as to assess the effect of resveratrol on phenylephrine- (PHE-) induced contraction of vascular smooth muscle cells (VSMCs). Moreover, we attempt to define the dependence of contraction mechanisms on endothelial NO synthase. Experiments were performed on Wistar rats (*n* = 17) pretreated with resveratrol (4 weeks; 10 mg/kg* p.o.*) or placebo. Serum BDNF levels were quantified after 2 and 4 weeks of treatment with ELISA. Contraction force was measured on isolated and perfused tail arteries as the increase of perfusion pressure with a constant flow. Values of serum BNDF in week 0 were 1.18 ± 0.12 ng/mL (treated) and 1.17 ± 0.13 ng/mL (control) (*p* = ns). After 2 weeks of treatment, BDNF in the treatment group was higher than in controls, 1.52 ± 0.23 ng/mL and 1.24 ± 0.13 ng/mL, respectively. (*p* = 0.02) Following 4 weeks of treatment, BDNF values were higher in the resveratrol group compared to control 1.64 ± 0.31 ng/mL and 1.32 ± 0.26 ng/mL, respectively (*p* = 0.031). EC_50_ values obtained for PHE in resveratrol pretreated arteries were significantly higher than controls (5.33 ± 1.7 × 10^−7 ^M/L versus 4.53 ± 1.2 × 10^−8 ^M/L, *p* < 0.05). These results show a significant increase in BDNF concentration in the resveratrol pretreated group. The reactivity of resistant arteries was significantly reduced for resveratrol pretreated vessels and this effect was partially NOS-3 independent.

## 1. Introduction

Resveratrol (3,4′,5-trihydroxy-trans-stilbene), a natural nonflavonoid polyphenol found in grapes and red wine, has antineuroinflammatory properties and is known to be neuroprotective in ischemia, seizure, and neurodegenerative diseases [1, 2]. Resveratrol has the ability to reduce inflammation by scavenging reactive oxygen species (ROS) [3], activating Sirtuin-1- (Sirt-1-) dependent anti-inflammatory pathways [1], interfering with Toll-like receptor 4/nuclear factor *κ*-light-chain enhancer of activated B cells/signal transducer and activator of transcription (TLR4/NF-*κ*B/STAT) signaling cascade with resultant decrease in cytokine production from activated microglia [4]. The aforementioned polyphenol, due to the anti-inflammatory properties, may be a potential therapeutic option in the treatment of neurodegenerative diseases, as the efficacy of resveratrol had been proven in preclinical and in vitro trials [5]. Beneficial effect of resveratrol treatment has been shown in disorders such as Alzheimer's disease, Parkinson's disease, Huntington's disease, amyotrophic lateral sclerosis [6], and vascular dementia [7]. Nevertheless, although numerous in vitro and animal model trials have shown the effectiveness of resveratrol, clinical trials are unfulfilled or data from them is not yet provided.

The brain-derived neurotrophic factor (BDNF), a member of neurotrophin family protein, plays an important role in survival and differentiation of neural structures during the development of the nervous system [8]. The main mechanism of action of BDNF is a ligand-like specific binding to tropomyosin-related kinase B (TrkB) via which it mediates different neurotropic signaling and increases brain plasticity. BDNF is involved in the differentiation and maturation of nerve cells in the central nervous system. The aforementioned neurotrophin is also associated with increased ratio of growth, formation of new neuronal connections, and nerve branching, as well as induction of synaptic transmission [9–11]. The decrease of serum BDNF levels is associated with aggravation and poor outcome in neurodegenerative diseases [12–15]. According to rat model study, BDNF mRNA is widely expressed in central nervous system; however, the biggest concentrations of the aforementioned neurotrophin are present in hippocampal mossy fibers [16]. In neurons, BDNF is localized to nerve terminals in secretory granules where it is cleaved by prohormone convertase 1 (PC1) [17]. After stimulation, BDNF is subdued, an axonal transport that proceeds from presynaptic nerve terminals in anterograde manner [16, 18]. Release of BNDF is associated with nerve depolarization and intra- and extracellular concentrations of Ca^2+^ ions [19]. The main source of circulating BDNF, both at rest and during exercises, is brain. Release of the neurotrophin from cerebral vascular endothelium is the result of local hypoxic stress and is mediated by *β*-1 Integrins and Integrin-Linked Kinase (ILK). Increased activity of brain results in elevated BDNF concentrations that can be measurable in internal jugular vein [20, 21].

The function and lowered concentration in diseases associated with neuronal damage, taken together, point out that physiological serum levels of BDNF contribute to preservation of proper functioning of the neurons located in central nervous system. Although in vitro studies confirm that BDNF has therapeutic potential in the treatment of neurodegenerative disorders, clinical trials present disappointing results [22]. The use of exogenous BDNF as a therapeutic agent is limited due to properties of the protein [12, 23]. The biggest challenge is to effectively deliver BDNF to predetermined target structures of the nervous system [24]. Plasma half-life of the protein in rats is less than one minute; moreover, BNDF is characterized by its poor ability to cross the blood-brain barrier and poor brain intraparenchymal penetration [22, 23]. Taking together these facts, a more efficient therapeutic option may be used to indirectly increase endogenous BDNF release stimulation using antineuroinflammatory agents.

Other potentially beneficial mechanism of resveratrol action in the prevention of neurodegeneration and cognitive impairment is the ability of polyphenol to reduce vascular smooth muscle cell (VSMC) contractility. Cognitive impairment and dementia are characterized by defective cerebrovascular blood flow mechanisms which are considered to be a significant element in its pathogenesis. Moreover, Araya et al. state that cerebrovascular abnormalities, especially in cerebral microvessels, potentially lead to neuronal dysfunction and cognitive impairment [25, 26]. Chronic systemic diseases are thought to impair vasorelaxation, with the consequence that cerebral blood flow is diminished, as they are associated with the production of proinflammatory cytokines and reactive oxygen species (ROS) [26]. Maintenance of cerebral blood flow at both stable and adequate levels seems to be a potential target in the pharmacological prevention of neurodegeneration.

A positive relationship between resveratrol and increased BDNF mRNA expression has been observed in recent studies [27]. However, to our knowledge, there are no previous investigations evaluating the effect of resveratrol administration on serum BDNF concentrations. We hypothesize that the administration of resveratrol may not only have antineuroinflammatory effects but also indirectly contribute to BDNF-induced neuroprotection by increasing concentrations of the protein. Following the fact that serum BDNF concentrations reflect brain-tissue BDNF level [28], in our study, we decided to define the relationship between serum BNDF and long-term oral resveratrol administration in the rat model. Another objective was the assessment of the mechanisms underlying the resveratrol-induced relaxation of VSMCs and their dependence on endothelial NO synthase (NOS-3).

## 2. Materials and Methods

### 2.1. Animals

Institutional Animal Ethics Committee approval was acquired before the beginning of the study (n°.: 17/2015). Male Wistar rats (*n* = 17) weighing 250–350 grams were selected for the study. The rats were maintained in 12 h light and dark cycles in a temperature and humidity controlled environment. Ad libitum access to food and drinking water during the day and night was provided. The rats were randomised and qualified to treatment and control groups (*n* = 8). The treatment group was given resveratrol orally for a period of 4 weeks, whereas controls were administered placebo. Blood sampling for biochemical tests was performed via catheters placed in the femoral vein. Pharmacometrics were performed after 4 weeks of resveratrol pretreatment on isolated rat tail arteries, as it is a recognized model for arterial resistance [29]. Before tail artery dissection, rats were anesthetized with intraperitoneal urethane 120 mg/kg. Sacrifice was performed by cervical vertebrae dislocation. The trial was conducted according to United States NIH guidelines, and care was taken to handle the rats in a humane manner.

### 2.2. Drugs and Solutions

The study drug (resveratrol) was administered orally through gastric intubation. The dose used in the study was 10 mg/kg. Reagents used during pharmacometric tests were L-NAME [(N-nitro-L-arginine-methyl ester)] 10–5 M/L and Krebs-Henseleit solution (NaCl (71,8 mM), KCl (4,7 mM), CaCl_2_ (1,7 mM), NaHCO_3_ (28,4 mM), MgSO_4_ (2,4 mM), KH_2_PO_4_ (1,2 mM), and glucose (11,1 mM)). All reagents were purchased from Sigma-Aldrich Chemical Company (Poznan, Poland).

### 2.3. Study Design and Conduction

The trial was divided into two experimental phases. In the first phase, resveratrol solution was administered to rats (10 mg/kg daily) for 4 weeks or the same amount of water. Before administration of resveratrol or vehicle, a blood sample was obtained which served as the control. Blood sampling was also performed after 2 and 4 weeks from the initiation of the trial. Blood was collected in sample tubes containing clotting activator and centrifuged for 15 min at 160 grams. The supernatant was stored −86°C. Serum BDNF levels in control, after 2 weeks and 4 weeks of treatment, were quantified using a commercially available ELISA kit (Shanghai Sunred Biological Technology Co., Ltd).

The pharmacometric analysis design was analogous to previously performed trials [30]. 2.5 to 3.0 cm long segments of rat tail arteries without significant branching were dissected from surrounding tissues and placed in 20 mL container. A cannula was introduced through the proximal part of the artery and connected to a perfusion device. The distal part was weighed with a 500 mg weight. During the initial part of the assay, the vessel was stabilized in oxygenated Krebs-Henseleit solution, at 37.0°C and pH 7.4. Perfusion was achieved using peristaltic pump with flow gradually increased from 0.25 to 1.0 mL/min, until a perfusion pressure within 2–4 kPa was achieved. Contractions of the vessels induced by phenylephrine, a full *α*1-adrenergic receptor agonist, in the control conditions as well as in the presence of L-NAME, were measured with increasing perfusion pressures. The addition of L-NAME is due to the results of previous trials which demonstrates it to be an inhibitor of NOS-3. L-NAME is the prodrug synthesized to L-NOARG which has the ability to inhibit NOS with IC_50_ of 1.4 *μ*M [31].

### 2.4. Data Analysis and Statistical Procedures

Statistical analysis was performed using the STATISTICA12 software. The Shapiro-Wilk test was used to determine the normal distribution of investigated variables. For comparing values of two following measurements, the ANOVA test was used. Results were presented as mean values ± standard deviations. *p* values < 0.05 were considered significant. Concentration-response curves (CRCs) were calculated according to the van Rossum method [32]. The maximal response of tissue (*E*_max_) was calculated as a percent of the maximal response for PHE. Half maximal effective concentration (EC_50_) was estimated using classical pharmacologic methods with pD2 the negative logarithm of the EC_50_. We used the number of the CRC and *E*_max_ in all calculations estimating the statistical significance.

## 3. Results

### 3.1. The Effect of Resveratrol on Serum BDNF Concentration Levels

**Figure 1:**
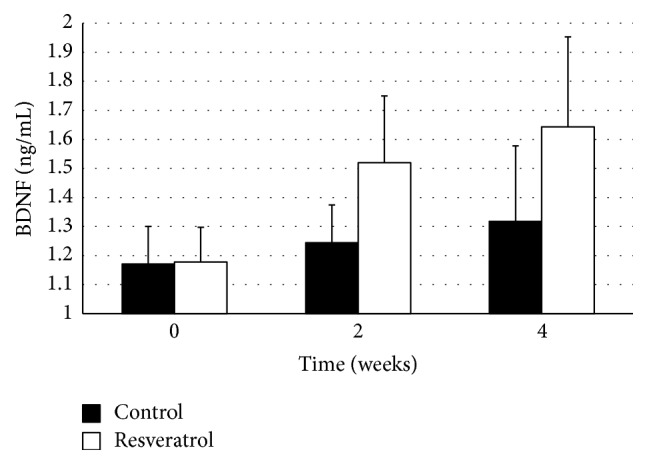
Serum BDNF concentration before resveratrol administration and after 2 weeks and 4 weeks of resveratrol pretreatment. “2nd week” and “4th week” bars represent values of serum BDNF after 2 and 4 weeks of resveratrol administration, respectively. Whiskers display ± standard deviations. Values presented in nanograms per milliliter. BDNF: serum concentration of brain-derived neurotrophic factor.

In the first part of the experiment, we compared BDNF concentrations between resveratrol and control groups immediately before administration of the first dose and after 2 weeks and 4 weeks of treatment. Initial values of serum BDNF in control and resveratrol groups were 1.17 ± 0.13 ng/mL and 1.18 ± 0.12 ng/mL, respectively (*p* = ns). After 2 weeks of treatment, there was observed a statistically significant increase in serum BDNF concentrations in comparison to treated group at time 0 (*p* = 0.004). Serum BDNF levels in resveratrol and control groups were 1.52 ± 0.23 ng/mL and 1.24 ± 0.13 ng/mL, respectively, with values in treatment group significantly higher in comparison to controls (*p* = 0.02). After 4 weeks of resveratrol treatment, serum BDNF concentration was significantly increased (*p* = 0.031) in comparison to control group; however, the increase was nonsignificant in comparison to the 2-week resveratrol group. The values in resveratrol and control groups were 1.64 ± 0.31 ng/mL and 1.32 ± 0.26 ng/mL, respectively (Figure 1).

### 3.2. Effect of Resveratrol on Vascular Smooth Muscle Cell Contractility

**Figure 2:**
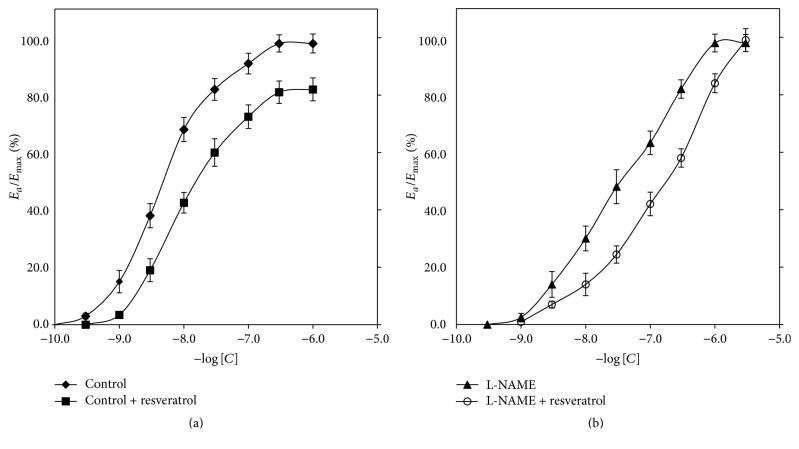
Concentration-response curves in arteries with vascular endothelium obtained for phenylephrine in control and the resveratrol pretreated group (a); solely L-NAME and L-NAME with the presence of resveratrol (b). “Control” group serves as the control for “resveratrol” group (a), whereas “L-NAME” group serves as the control for “L-NAME + Resveratrol” group (b). Points and whiskers display mean values ± standard deviations. “Control” curve derived to represent a control curve for resveratrol. *E*_*a*_/*E*_max_: % of maximal response; ^*∗*^a value of *p* < 0.05 when comparing the control curve for points of effect between 20% and 80% of the maximal response.

CRCs calculated for arteries with endothelium derived from rats after 4 weeks of treatment with resveratrol were shifted to the right of control with the maximal response decrease of 16% (*p* < 0.001) (Table 1). EC_50_ value of arteries with vascular endothelium calculated for PHE in rats after 4-week resveratrol administration was 5.33 ± 1.7 × 10^-7 ^M/L, whereas control value for PHE was 4.53 ± 1.2 × 10^-8 ^M/L and the increase was statistically significant (*p* < 0.05). Corresponding values are presented in Figure 2.

CRCs calculated for PHE in resveratrol-treated rat arteries, in the presence of L-NAME, were shifted to the right compared to groups without L-NAME, with a statistically significant increase of EC_50_ (*p* < 0.05). Corresponding pD2 values are presented in Figure 2. EC_50_ values for both resveratrol + L-NAME and L-NAME groups were 4.13 (±2.12) × 10^-8 ^M/L and 3.42 (±0.85) × 10^-7 ^M/L, respectively. The increase of EC_50_ in resveratrol + L-NAME group was statistically significant compared to solely L-NAME (*p* < 0.05). There was no significant difference in maximal response between resveratrol + L-NAME and L-NAME groups (Table 1).

### 3.3. Effect of Resveratrol on Perfusion Pressure in the Presence and Absence of L-NAME

**Figure 3:**
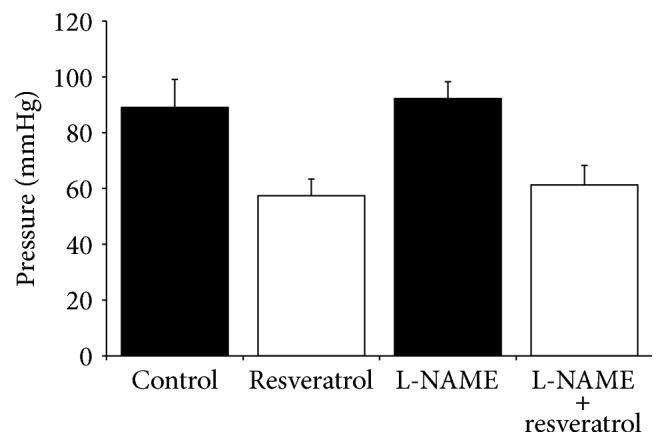
Perfusion pressures in arteries in the resveratrol pretreated group in the presence of L-NAME in comparison to control and L-NAME. Control represents values for PHE and serves as the control for control + resveratrol, whilst L-NAME is a control for L-NAME + resveratrol. Pressure values are represented by millimeters mercury.

A significant reduction in the perfusion pressure in resveratrol pretreated rat arteries was noted in comparison to control. Perfusion pressures in control and resveratrol groups were 89.1 ± 10 mmHg and 57.4 ± 6 mmHg, respectively (*p* < 0.05). In the presence of L-NAME, an analogous significant reduction in perfusion pressure was seen in resveratrol pretreated rat arteries, with values of 92.25 ± 10 mmHg for L-NAME control group and 61,3 ± 7 mmHg for resveratrol/L-NAME group (*p* < 0.05). Reduction of perfusion pressures was also compared between resveratrol and resveratrol/L-NAME groups, with results not showing any statistically significant difference in perfusion pressure between both groups (Figure 3).

## 4. Discussion

Resveratrol is polyphenolic compound found in the seeds and skins of grapes, red wine, mulberries, peanuts, and rhubarb which is considered to have a wide range of beneficial properties, especially related to cardiovascular diseases. Additionally, recent studies have elucidated neuroprotective features of resveratrol and their potential mechanisms [2]. In this trial, we analyzed the effect of resveratrol on serum BDNF concentration and VSMCs contractility.

The results we obtained indicate that resveratrol influences serum BDNF concentrations. After 4 weeks of resveratrol administration in our rat model, we noticed a significant elevation of BDNF concentration in serum. Induction of BDNF expression in brain structures after administration of naturally existing plant-derived polyphenols was previously described by Jeon et al. [33]. Although our study uses serum BNDF concentration, we deem that the results correspond with brain levels of BDNF, as resveratrol crosses the blood-brain barrier and the correlation between serum and brain BDNF levels has been verified [28, 34]. Zhang et al. found that resveratrol induces BDNF release from astroglia in rat primary astroglia-enriched cultures suggesting that resveratrol administration may be more efficient than direct treatment with neurotrophic factors [35].

BDNF seems to be associated with neurovascular coupling. Potentially, the aforementioned neurotrophin constitutes the link in maintaining cerebral blood flow in response to hypoxic stress. Guo et al. suggest that BDNF seems to be playing a dominant role in the neurovascular unit of brain. Their results confirm that cerebrovascular endothelium can secrete potent neuroprotective agents [21].

The mechanism of BDNF upregulation by resveratrol is not fully elucidated. According to Goggi et al., the release of BDNF depends on the concentrations of both extracellular and intracellular calcium. They have also noticed that BDNF release is associated with the activation of IP_3_ mediated Ca^2+^ release from intracellular stores. BDNF was also modulated by receptors coupled to adenylate cyclase. Resveratrol is potent inhibitor of phosphodiesterase-1 (PDE-1) responsible for metabolism of cAMP; moreover, resveratrol presents the ability to modulate Ca^2+^ levels [19, 36, 37]. Resveratrol is also known as a Sirt-1 activator [1] and may upregulate BDNF and TrkB via Sirt-1-dependent pathways [38]. Another probable mechanism is activation of the CREB and ERK1/2 signaling pathways which result in an increased production of neurotropic factors [35]. It was noted that the BDNF gene contains a CRE, which binds phosphorylated CREB, thereby enhancing transcription, potentially supporting this hypothesis [39]. Subsequently, agents like resveratrol that induce the expression of BDNF are believed to reproduce the biological effects of the neurotrophin.

In the second part of our study, we studied the ability of resveratrol to reduce VSMC contractility. We took into consideration the fact that, in the resveratrol-treated group in the presence of NOS-3 inhibitor (L-NAME), there was a significant reduction of phenylephrine-induced contractility in comparison to control group with L-NAME. Our finding was that nonsignificant difference in both EC_50_ and perfusion pressures in resveratrol-treated groups with and without NOS-3 inhibitor combined confirms that the effect of resveratrol is NOS-3-independent. The main product of NOS-3 activity, nitrogen oxide (NO), is a potent vasorelaxant as it activates soluble guanylyl cyclase leading to consequent accumulation of guanosine 3′:5′-cyclic monophosphate (cGMP) in smooth muscle cells [31]. cGMP acts through protein-kinase G with subsequent inactivation of L-type calcium channels [40]. Resveratrol has the ability to induce NOS-3 in both a direct and indirect manner through AMPK, SIRT1, and Nrf2 pathways and, as a result, it positively affects vasorelaxation in cerebral arteries [41].

Results presented in our study seem to be consistent with those obtained in previous trials. Leblais et al. state that resveratrol may directly act on VSMCs promoting pulmonary artery relaxation via different mechanisms including the following: induction of guanylyl cyclase, inhibition of protein-kinase C, activation of smooth muscle K^+^ channels, or acting via Ca^2+^ [42]. Kline and Karpinski suggest that resveratrol acts on VSMCs by direct blocking of L-type calcium channel and inhibiting intracellular Ca^2+^ release [36]. There are other potential mechanisms of action of resveratrol on VSMCs described.

Direct reduction of VSMC contractility by resveratrol may be an important mechanism in neuroprotection, as pathogenesis of neurodegenerative diseases is vasoconstriction-mediated [43]. The exact pathogenesis of neurodegenerative diseases and cognitive impairment is not fully elucidated; however, in recent clinical practice, it is generally accepted that maintaining constant cerebral blood flow may have a beneficial effect on cognitive abilities and prevention of neuronal damage. Chronic inflammation and oxidative stress, inseparable elements of neurodegenerative diseases, may lead to dysregulation of various mechanisms controlling cerebral blood flow including prostacyclin-, endothelium-derived hyperpolarizing factor- (EDHF-), and NO-dependent vasodilatation, which is impaired under oxidative stress conditions [25, 43]. Following this, the fact that resveratrol not only acts through NOS-3 but also via different mechanisms may potentially preserve cerebral blood flow at high levels providing neuronal protection in chronic neuroinflammation.

Our study has several limitations. First, as in all experimental studies, the relevance of the results needs verification in the clinical setting, as a human study comparing the effect of resveratrol on BDNF levels and cerebrovascular resistance could only provide a definitive answer. Second, in-depth determination of the mechanism of resveratrol influence on neurovascular coupling warrants further investigation including behavioral examination and/or epigenetic testing. Finally, the determination of equivalent drug exposures in the rat model versus clinically observed was approximated only.

In summary, resveratrol increases BDNF serum concentrations which, according to literature, reflects an increase of BDNF in brain parenchyma. We have also shown that resveratrol reduces the contractility of resistance arteries via NOS-3-independent mechanisms, highlighting the need for further investigations in maintaining stable cerebral blood flow.

## Figures and Tables

**Table 1:** Maximal relative response for phenylephrine in relation to the absence and presence of L-NAME in resveratrol treated and control groups.

	*n* ^1^	*E*_max_ [%]^2^	*p*
Control, PHE (10 *μ*M/L)	8	98.0 ± 3.3	—
Resveratrol pretreated rats, PHE (10 *μ*M/L)	16	82.0 ± 2.0	**p** < **0.001**^a^
Control (PHE) 10 *μ*M/L + L-NAME (10^−5 ^M/L)	8	98.0 ± 3.0	—
Resveratrol pretreated rats + L-NAME (10^−5 ^M/L)	12	99.0 ± 4.0	**p** = **ns**^b^

^1^Number of concentration-response curves used for calculations. ^2^*E*_max_: calculated as a percent of maximal response for PHE; ^a^*p* value calculated versus controls; ^b^*p* value calculated versus control (PHE) 10 *μ*M/L + L-NAME. PHE: phenylephrine.
